# Disturbances of Mitochondrial Functions and Oxidative Stress Induction in Methylmalonic and Propionic Acidemias: A Critical Role for Metabolite Accumulation

**DOI:** 10.3390/ijms27188379

**Published:** 2026-09-20

**Authors:** Alexandre Umpierrez Amaral, Moacir Wajner

**Affiliations:** 1 Programa de Pós-Graduação em Atenção Integral à Saúde (UNICRUZ/URI-Erechim/UNIJUÍ), Universidade Regional Integrada do Alto Uruguai e das Missões, Erechim CEP 99709-910, RS, Brazil; 2 Programa de Pós-Graduação em Ciências Biológicas: Bioquímica, Departamento de Bioquímica, Instituto de Ciências Básicas da Saúde, Universidade Federal do Rio Grande do Sul, Porto Alegre CEP 90035-003, RS, Brazil; mwajner@ufrgs.br; 3 Serviço de Genética Médica, Hospital de Clínicas de Porto Alegre, Porto Alegre CEP 90035-903, RS, Brazil

**Keywords:** methylmalonic acidemia, propionic acidemia, organic acids, mitochondrial dysfunction, oxidative stress

## Abstract

Methylmalonic and propionic acidemias are inherited disorders of propionyl-CoA catabolism characterized by deficient activity of L-methylmalonyl-CoA mutase and propionyl-CoA carboxylase, respectively. They lead to metabolite accumulation in tissues and biological fluids, including methylmalonic, propionic, 3-hydroxypropionic, 2-methylcitric and maleic acids. Affected patients develop multi-systemic symptoms, with predominant neurological manifestations. Although current therapy based on dietary protein restriction significantly decreases mortality and morbidity, it is still insufficient to prevent long-term complications in most patients. The pathogenesis of methylmalonic and propionic acidemias has been investigated in recent decades using chemically induced in vivo models, genetic animal models, in vitro models and tissues and biological fluids from patients. Although the precise mechanisms responsible for the clinical manifestations in these disorders remain under debate, biomarkers of mitochondrial dysfunction and oxidative stress have been consistently described in tissues from affected patients and genetic murine models. Notably, growing evidence indicates that the organic acids accumulating in these disorders, which are formed within mitochondria, compromise mitochondrial functions through multiple mechanisms, disrupting bioenergetics, quality control, calcium homeostasis and redox balance, ultimately leading to cell death. This review discusses pathomechanisms of mitotoxicity caused by the major organic acids accumulating in methylmalonic and propionic acidemias from observations taken from patients and animal models.

## 1. Introduction

**Figure 1:**
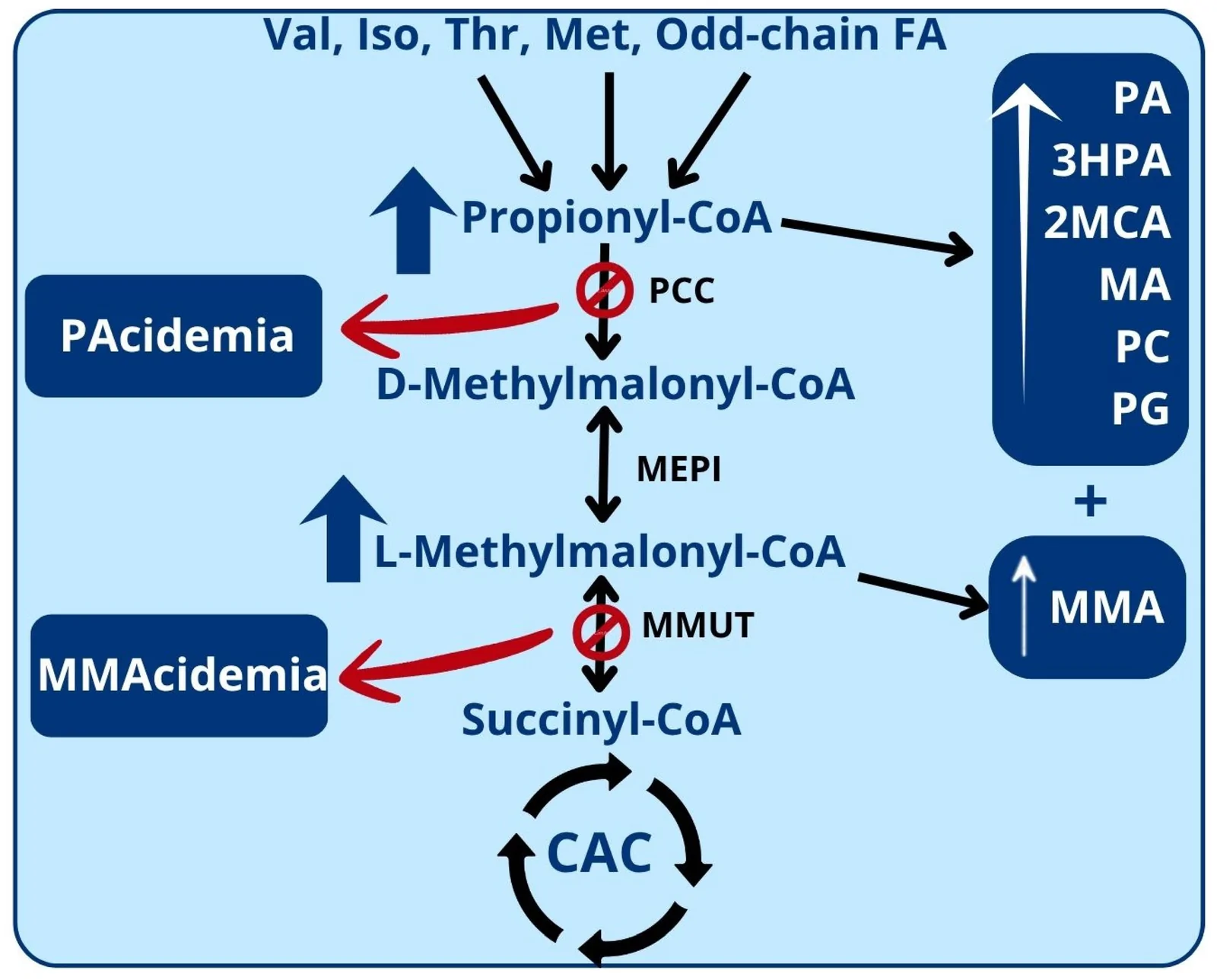
Blockage of catabolic pathways leading to metabolite accumulation in biological fluids and tissues of patients affected by PAcidemia and MMAcidemia. Propionyl-CoA is produced from the catabolism of valine (Val), isoleucine (Iso), threonine (Thr), methionine (Met) and odd-chain fatty acids (odd-chain FA). Propionic acidemia (PAcidemia) is caused by deficient activity of propionyl-CoA carboxylase (PCC), leading to accumulation of the propionyl-CoA-derived metabolites propionic acid (PA), 3-hydroxypropionic acid (3HPA), 2-methylcitric acid (2MCA), maleic acid (MA), propionylcarnitine (PC) and propionylglycine (PG). Methylmalonic acidemia (MMAcidemia) is caused by deficient activity of methylmalonyl-CoA mutase (MMUT), leading to predominant accumulation of methylmalonic acid (MMA) and the propionyl-CoA-derived 2MCA. CAC: citric acid cycle; MEPI: methylmalonyl-CoA epimerase. Figure created with Canva web application (Canva Pty Ltd., Sydney, Australia).

Methylmalonic acidemia (MMAcidemia) and propionic acidemia (PAcidemia) are inherited metabolic disorders of propionyl-CoA catabolism, derived from degradation of the amino acids isoleucine, methionine, threonine, and valine, and the β-oxidation of odd-chain fatty acids. MMAcidemia is caused by mutations in the gene *MMUT* encoding methylmalonyl-CoA mutase (OMIM 609058), mostly resulting in accumulation of mainly methylmalonic acid (MMA) and 2-methylcitric acid (2MCA) (Figure 1). Numerous pathogenic variants have been identified in the *MMUT* gene. The most common are c.322C>T (p.Arg108Cys), c.655A>T (p.Asn219Tyr) and c.1106G>A (p.Arg369His), which lead to a complete enzyme deficiency (*mut*^0^), as well as c.2150G>T (p.Gly717Val), which causes partial enzyme deficiency (*mut*^−^). Patients usually present with acute encephalopathy in the first months of life, as well as progressive neurologic deterioration, seizures, hypotonia, dystonia, hypertonia, ataxia, psychomotor delay and intellectual disability. Severe kidney damage occurs as the disease progresses [1].

PAcidemia is due to a genetic deficiency of propionyl-CoA carboxylase (OMIM 606054), the upstream enzyme in the catabolism of propionyl-CoA, causing predominant accumulation of propionic acid (PA), 3-hydroxypropionic acid (3HPA) and 2MCA. Maleic acid (MA) also accumulates in PAcidemia but to a lesser degree (Figure 1). The most common pathogenic variant found in the *PCCA* gene is c.425G>A (p.Gly142Asp), leading to a severe phenotype. In the *PCCB* gene, the most common variants include c.335G>A (p.Gly112Asp), c.1534C>T (p.Arg512Cys) and c.1556T>C (p.Leu519Pro), which are associated with a severe phenotype, and c.1304T>C (p.Tyr435Cys), which leads to a mild phenotype. Affected patients manifest with acute encephalopathy and severe neurological symptoms including seizures, coma, movement disorders and intellectual disability, accompanied by life-threatening cardiac complications. Some of them may also develop long-term kidney disease over time [2].

Diagnosis of PAcidemia is generally based on the detection of high amounts of PA, 3HPA, 2MCA and propionylglycine (PG) in urine, as well as propionylcarnitine in the blood [2]. Accumulation and high urinary excretion of these compounds, and particularly MMA, are found in MMAcidemia [3,4,5]. Treatment for MMAcidemia and PAcidemia fundamentally relies on restriction of protein and specific amino acids, and supplementation of L-carnitine, micronutrients and vitamins [6,7]. Although current treatment significantly decreases mortality, it does not prevent most long-term systemic and neurological complications. Patients with both disorders who do not respond to these therapeutic strategies undergo liver transplantation, which significantly reduces the accumulating metabolites, improving their quality of life. However, this therapeutic approach must be employed with caution, as it is considered a high-risk surgery and neurological improvement after transplantation is limited [8].

Clinical symptomatology commonly worsens during acute metabolic distress generally triggered by recurrent infections, vaccinations, or other metabolic stressors, and is attributed to increased production of the accumulating metabolites due to accelerated proteolysis. Preventing these episodes by maintaining the affected patients under rigorous metabolic control is critical for treatment efficacy, reinforcing that the toxicity of the accumulating metabolites is the main determinant of their symptoms, characterizing these disorders as intoxicating diseases. Although the precise underlying mechanisms of tissue damage in MMAcidemia and PAcidemia are still under debate and need to be further elucidated [9,10,11], the available evidence supports the hypothesis that the accumulating organic acids are toxic to mitochondrial functions, impairing cellular functions and promoting cell death. In this particular, multiple mechanisms of mitotoxicity of these compounds in MMAcidemia and PAcidemia have been described using different experimental approaches.

This review will summarize the current insights obtained from human and animal data indicating that mitochondrial dysfunction following metabolite accumulation is mainly involved in the pathogenesis of MMAcidemia and PAcidemia, through multiple underlying mechanisms leading to impaired mitochondrial bioenergetics, calcium homeostasis, quality control and redox balance.

## 2. Mitochondrial Functions

Mitochondria are central hubs of cellular metabolism, supplying most ATP required by high-energy-demanding tissues such as the brain, heart, skeletal muscle, kidney and liver [12]. Acetyl-CoA molecules derived from glucose, fatty acids, amino acids, and ketone body oxidation converge into the citric acid cycle (CAC) to generate NADH and FADH_2_, which deliver electrons to the respiratory chain. Electron flow drives proton translocation across the inner mitochondrial membrane, establishing the electrochemical gradient that sustains ATP synthesis through oxidative phosphorylation [12]. Efficient ATP generation requires proper substrate supply, intact transport systems, mitochondrial membrane potential (ΔΨm) and ultrastructure. Creatine kinase (CK) isoforms further support energy homeostasis by enabling rapid phosphate transfer between phosphocreatine and ADP, contributing to intracellular energy buffering [13].

Beyond energy production, mitochondria are critical regulators of intracellular calcium dynamics. Calcium enters the matrix through the mitochondrial calcium uniporter and is extruded mainly through Na^+^/Ca^2+^ and H^+^/Ca^2+^ exchangers. Mitochondria–endoplasmic reticulum (ER) contact sites generate high-calcium microdomains required for efficient mitochondrial uptake, linking calcium signaling to metabolic activation and survival [14,15]. Excessive matrix calcium, however, can trigger mitochondrial permeability transition (mPT) pore opening, causing membrane depolarization, inhibition of ATP synthesis, organelle swelling, and release of key metabolites to the cytosol. These events lead to apoptosis or necrosis and contribute to neurodegenerative, cardiovascular, hepatic, renal, and muscular pathologies [16].

Mitochondria are also major sources of reactive oxygen and nitrogen species (ROS and RNS). Electron leakage from the respiratory chain generates superoxide, while additional ROS arise from dehydrogenases, monoamine oxidases, and cytochrome P450 enzymes. Mitochondrial nitric oxide production may yield peroxynitrite, a potent oxidant implicated in mitochondrial injury [17]. Although physiological ROS and RNS participate in signaling, immune modulation, and vascular regulation, excessive formation causes oxidative damage to lipids, proteins, and nucleic acids. Antioxidant defenses, including reduced glutathione (GSH), superoxide dismutase (SOD), catalase, glutathione peroxidase (GPx), glutathione reductase (GR), glutathione S-transferase (GST) and peroxiredoxins, are responsible for neutralizing excessive reactive species generation and keep the redox balance. Of note, these enzymes differ in their subcellular distribution: Mn-SOD (SOD2) and peroxiredoxins 3 and 5 are predominantly mitochondrial, whereas Cu/Zn-SOD (SOD1), catalase and most GST isoforms are mainly cytosolic, with catalase largely peroxisomal. On the other hand, GPx and GR are present in both compartments. Thus, mitochondrial H_2_O_2_ detoxification relies primarily on GPx and peroxiredoxins, underscoring the mitochondrial relevance of this antioxidant network [18]. In this context, the NADPH pool, which is largely maintained by the cytosolic pentose phosphate pathway and by the activities of the mitochondrial enzymes isocitrate dehydrogenase, glutamate dehydrogenase, and nicotinamide nucleotide transhydrogenase, is critical for regenerating GSH levels and supporting NADPH-dependent enzymes, such as GPx [19].

Mitochondrial functions depend on quality control mechanisms, which are responsible for monitoring organelle integrity and function, as well as for repairing or eliminating damaged mitochondria. In this context, fusion mediated by optic atrophy 1 (OPA1), mitofusin 1 (Mfn1), and mitofusin 2 (Mfn2), supports mitochondrial bioenergetics and respiratory maintenance, whereas dynamin-related protein 1 (Drp1)-dependent fission enables segregation of damaged mitochondrial segments and facilitates mitophagy. Mitochondrial biogenesis, coordinated by peroxisome proliferator-activated receptor gamma coactivator 1-α (PGC1-α) and sirtuin 1 (SIRT1), and selective removal of dysfunctional mitochondria via PTEN-induced putative kinase 1 (PINK1)–parkin signaling (mitophagy), collectively preserve mitochondrial quantity and functionality [20,21]. Impairments of these processes compromise bioenergetics, calcium handling, and cellular stress adaptation, contributing to the pathogenesis of metabolic, neuromuscular, hepatic, cardiovascular, neurodegenerative, and neoplasic diseases [22,23].

Together, these interconnected pathways highlight the central role of mitochondria in sustaining cellular physiology and demonstrate how disturbances in mitochondrial function drive disease progression.

## 3. Evidence of Mitochondrial Dysfunction: Insights from Patients and Genetic Models of MMAcidemia and PAcidemia

Table 1 and Table 2 summarize the biochemical evidence of mitochondrial dysfunction in patients and genetic knockout animal models of MMAcidemia and PAcidemia.

### 3.1. Bioenergetics Dysfunction

In the available human observations in patients with PAcidemia and MMAcidemia, previous reports reveal mitochondrial abnormalities in skeletal muscle [24] and renal epithelial cells [25] from PAcidemia patients, as well as in livers [26,27,28] and kidneys [29,30,31] from patients with MMAcidemia, as detailed in Table 1.

Furthermore, lactic acidosis and lactic aciduria were consistently reported in PAcidemia [2,32,33,34,35,36,37] and MMAcidemia [1,32,34,37,38], particularly during episodes of metabolic decompensation. Elevated lactate levels were also reported in cerebrospinal fluid [39], the globus pallidus and white matter of a metabolically decompensated patient with MMAcidemia [40]. Notably, increased lactate production is a frequent finding reflecting impaired mitochondrial bioenergetics, implying lactic acidosis as a biochemical hallmark of mitochondrial dysfunction [41,42].

Other observations of mitochondrial dysfunction were observed in these disorders, including decreased plasma [43] and myocardial levels [44] of coenzyme Q_10_ (CoQ_10_), reduced myocardial free carnitine [45], and inhibition of respiratory chain activity in skeletal muscle [24,46], liver [27,46] and heart [46] biopsies of patients affected by PAcidemia. Decreases in pyruvate dehydrogenase (PDH) and α-ketoglutarate dehydrogenase (α-KGDH) activities, in addition to ATP and phosphocreatine levels, were also found in the skeletal muscle of these patients [24]. Moreover, alterations in CAC intermediate levels were found in renal epithelial cells from PAcidemia patients [25] and a decrease in mitochondrial respiration was associated with increased ROS generation in induced pluripotent stem cell (iPSC)-derived cardiomyocytes from a PAcidemia patient [47]. iPSC-derived cardiomyocytes from PAcidemia patients have also been shown to switch their energy metabolism from fatty acid oxidation to the less efficient glucose oxidation, implying a bioenergetic failure [48].

Patients with MMAcidemia also exhibit disturbances of CAC, respiratory chain complexes and oxidative phosphorylation in the liver [26,27,46] and kidney [46], together with decreased mitochondrial respiration [29,31] and reduced CoQ_10_ levels [49] in patient-derived cells. CAC disruption determined by changes in the CAC intermediate levels was also verified in renal epithelial cells from MMAcidemia patients [50]. Other findings including decreased cell viability, oxygen consumption, and ATP production were observed in kidney tubular epithelial cells from an MMAcidemia patient [51]. More recently, a reduction in the translocase of the outer mitochondrial membrane complex subunit 20 (TOMM20) content and ΔΨm were found in iPSC-derived neurons from MMAcidemia patients, indicating disruptive mitochondria [52].

Altered skeletal muscle mitochondrial energy metabolism has also been described in patients with these disorders, associated with decreased inorganic phosphate (Pi) levels in PAcidemia and reduced ATP content in MMAcidemia [53].

Experimental studies carried out in genetic animal models of these disorders further corroborate the human findings of mitochondrial dysfunction described in the affected patients. Thus, reduced respiratory chain complex activities were observed in the brain, liver, heart and skeletal muscle of the genetic mouse model of PAcidemia [54], as well as in the liver of the genetic murine model of MMAcidemia [26]. Decreased α-KGDH activity and abnormally shaped mitochondria were also found in the liver of the genetic murine model of MMAcidemia [55,56].

More recently, alterations in renal mitochondrial ultrastructure associated with damage to CAC-dependent energy generation were found in the genetic mouse model of PAcidemia [57]. Another study showed that the lethality of *PCCA* knockout in mice was reversed by simultaneously inhibiting pyruvate carboxylase, implying an important role for 2MCA systemic toxicity, since its formation arises from condensation of propionyl-CoA plus oxaloacetate catalyzed by this enzyme [58].

Furthermore, reduced mitochondrial oxidative phosphorylation capacity associated with a decreased lifespan was also demonstrated in propionyl-CoA carboxylase-mutant Caenorhabditis elegans [59]. In addition, altered mitochondrial morphology characterized by increased mitochondrial circularity with perturbed cristae organization was described in the kidney and liver of a zebrafish model for studying MMAcidemia [31].

### 3.2. Disruption of Calcium Homeostasis

A few studies reveal a disturbance of calcium homeostasis. Thus, cardiac dysfunction linked to lower systolic calcium release, impairment in the sarcoplasmic reticulum (SR) calcium load and decreased calcium re-uptake by SR-calcium ATPase was found in the genetic mouse model of PAcidemia [60]. Another study demonstrated disruption of calcium homeostasis associated with altered expression of proteins involved in ER stress, mitochondria–ER crosstalk and reduced oxygen consumption in iPSC-derived cardiomyocytes from a patient with PAcidemia [61].

### 3.3. Impairment of Mitochondrial Quality Control

Mitochondrial quality control represents a coordinated cellular network that maintains mitochondrial integrity, regulates energy metabolism, and prevents cell toxicity. It operates through three main pathways: mitochondrial dynamics (fusion and fission), mitophagy (selective degradation of damaged mitochondria), and mitochondrial biogenesis [62,63].

Impaired PINK1/Parkin-mediated mitophagy has been demonstrated in cultured kidney cells from MMAcidemia patients, leading to accumulation of damaged mitochondria with bioenergetics failure [31]. Mitochondrial mass was increased in renal tubular cells from PAcidemia patients and further augmented by high protein exposure, and this was associated with altered mitochondrial bioenergetics due to accumulation of CAC intermediates and silenced mitophagy [25].

Mitophagy was also disabled in renal epithelial cells from MMAcidemia patients, whereas mitochondrial dynamics were shifted towards fission (increased expression of Drp1, and decreased expression of OPA1 and PGC1α). These alterations were particularly relevant when cells were under high protein exposure [50].

Furthermore, changes in mitochondrial quality control were found in the kidney of the mouse model of PAcidemia. A significant reduction in the protein expression of PGC1α, OPA1, Mfn1, Mfn2 and PINK1, as well as increased expression of Drp1, indicate a clear tendency towards mitochondrial fission and impaired mitophagy [57].

### 3.4. Oxidative Stress

Although oxidative stress can be induced by several mechanisms, it is also linked to mitochondrial dysfunction, arising from impaired electron transport through the respiratory chain through increased electron leakage and excessive generation of reactive oxygen species [64,65].

Studies in individuals with disorders of propionate metabolism consistently show increased oxidative stress. Ribas et al. [66] and Atkuri et al. [67] reported elevated plasma concentrations of malondialdehyde (MDA), protein carbonyl, and decreased sulfhydryl groups in both MMAcidemia and PAcidemia. Changes in thiol-disulfide homeostasis were also recently reported in the plasma of these patients [68]. Urinary biomarkers further support these findings, with increased isoprostanes and dityrosine found at diagnosis and accompanied by reduced antioxidant capacity [69,70]. MDA and nitric oxide were also demonstrated to be increased in the serum of MMAcidemia patients, whereas GSH and SOD were decreased [71]. These alterations were shown to be more severe in untreated patients [72].

These mechanisms have been confirmed in patient-derived cells. Gallego-Villar et al. [73] found increased ROS levels and apoptosis in fibroblasts from a PAcidemia patient, whereas enhanced oxidative stress was described in renal tubular cells from patients [25]. Richard et al. [74] showed increased ROS, altered MnSOD expression, mitochondrial glycerol-3-phosphate dehydrogenase (GPDH)-dependent ROS production, and activation of mitochondrial caspase-mediated apoptosis in MMAcidemia fibroblasts. Additional evidence from blood and kidney cells of MMAcidemia patients confirms elevated mitochondrial ROS production [31] and impaired antioxidant capacity reflected by a reduced ratio of GSH to oxidized glutathione (GSSG) [50,75], reinforcing oxidative stress as a central contributor to disease pathophysiology. Decreased antioxidant capacity was also found in kidney tubular epithelial cells from a patient with MMAcidemia [51].

Biomarkers of oxidative damage, including isoprostanes (lipid peroxidation), di-tyrosine (protein oxidation), 8-hydroxy-2′-deoxyguanosine, 8-hydroxyguanosine, and 8-hydroxyguanine (DNA/RNA oxidation) were increased in the urine of MMAcidemia and PAcidemia patients [76]. MDA levels were also elevated and sulfhydryl content reduced in the plasma of these patients [72].

Oxidative stress is similarly increased in genetic models of PAcidemia and MMAcidemia. Gallego-Villar et al. [54] demonstrated elevated MDA, isoprostanes and ROS, along with reduced GPx expression in multiple tissues (brain, liver, skeletal muscle, and heart) from the mouse model of PAcidemia, indicating disruption of cellular redox balance. Oxidative stress induction was also verified in *PCCA* knockdown HL-1 cells (cardiac muscle cell line) and in the hearts of PAcidemia mice following prolonged exposure to PA [77]. Significant reductions in both GSH and GSSG levels were also observed in the liver of the MMAcidemia mutant mice, reflecting impaired antioxidant defenses [26].

**Table 1:** Mitochondrial dysfunction in patients with PAcidemia and MMAcidemia.

Organic Aciduria	Sample	Biochemical and Morphological Mitochondrial Alterations	References
PAcidemia	Blood	Lactic acidosis	[36]
		↓ CoQ_10_ levels	[43]
		↑ MDA levels	[66,71,72]
		↑ Carbonyl formation	[66,67]
		↓ Sulfhydryl content	[72]
		Changes in thiol-disulfide homeostasis	[68]
		↑ Nitric oxide	[71]
		↓ GSH and SOD activity	[71]
	Urine	↑ Isoprostanes and di-tyrosine	[69,76]
		↑ 8-hydroxy-2′-deoxyguanosine, 8-hydroxyguanosine, and 8-hydroxyguanine	[76]
		↓ Antioxidant capacity	[70]
	Liver	↓ Complex IV activity	[27]
		↓ Complex III and IV activities	[46]
	Skeletal muscle	Swollen mitochondria with disrupted cristae	[24]
		↓ Complex I, II, III and IV activities	[24]
		↓ Complex III and IV activities	[46]
		↓ PDH and α-KGDH activities	[24]
		↓ ATP and phosphocreatine	[24]
		↓ Pi levels	[53]
	Kidney	Altered mitochondrial morphology	[25]
		Disabled mitophagy	[25]
		Alterations in CAC intermediates	[25]
		↓ Oxygen consumption and ATP production	[51]
		↑ ROS levels	[25]
	Heart	↓ Complex III activity	[46]
		↓ CoQ_10_ levels	[44]
		↓ Free carnitine	[45]
	Fibroblasts	↑ ROS levels	[73]
	iPSC-derived cardiomyocytes	Disruption of calcium homeostasis	[61]
		Altered protein expression related to mitochondria–ER crosstalk	[61]
		↓ Mitochondrial respiration	[47]
		↑ ROS levels	[47]
		Switch their energy metabolism from fatty acid oxidation to the less efficient glucose oxidation	[49]
MMAcidemia	Blood	Lactic acidosis	[38]
		↑ MDA levels	[66,72]
		↑ Carbonyl formation	[66,67]
		↓ Sulfhydryl content	[72]
		↓ Ratio of GSH to GSSG	[75]
	Urine	↑ Isoprostanes and di-tyrosine	[69,76]
		↑ 8-hydroxy-2′-deoxyguanosine, 8-hydroxyguanosine, and 8-hydroxyguanine	[76]
		↓ Antioxidant capacity	[69]
	CSF	↑ Lactate levels	[39]
	Globus pallidus and white matter	↑ Lactate levels	[40]
	Fibroblasts	Alterations of mitochondrial morphology	[29]
		↑ Apoptosis through the mitochondrial/caspase-dependent pathway	[29]
		↓ Mitochondrial respiration	[29]
		↓ CoQ_10_ levels	[49]
		↑ ROS levels and Mn-SOD expression level	[74]
	Liver	Enlarged mitochondria	[28]
		Mitochondria with abnormal size and cristae conformation	[26]
		↓ Complex IV activity	[27]
		↓ Complex I, III, IV and V activities	[46]
		↓ Complex II-III and IV activities	[26]
	Skeletal muscle	↓ ATP content	[53]
	Kidney	↑ Fragmented mitochondria	[31]
		Impaired PINK1/Parkin–mediated mitophagy	[31]
		Disabled mitophagy	[50]
		↓ Complex I, II, III and IV activities	[46]
		↓ Mitochondrial respiration	[29,31]
		↓ Oxygen consumption and ATP production	[51]
		↓ ΔΨm	[31]
		Alterations in CAC intermediates	[50]
		↑ Expression of Drp1	[50]
		↓ Expression of OPA1 and PGC1α	[50]
		↑ ROS levels	[31]
		↓ Ratio of GSH to GSSG	[50]
	iPSC-derived neurons	↓ ΔΨm	[52]
		↓ TOMM20 content	

CoQ_10_: coenzyme Q_10_; CSF: cerebrospinal fluid; Drp1: dynamin-related protein 1; ER: endoplasmic reticulum; GSH: reduced glutathione; GSSG: oxidized glutathione; α-KGDH: α-ketoglutarate dehydrogenase; MDA: malondialdehyde; ΔΨm: mitochondrial membrane potential; MMAcidemia: methylmalonic acidemia; OPA1: optic atrophy 1; PAcidemia: propionic acidemia; PDH: pyruvate dehydrogenase; PGC1α: peroxisome proliferator-activated receptor gamma coactivator 1-α; Pi: inorganic phosphate; PINK1: PTEN-induced putative kinase 1; ROS: reactive oxygen species; SOD: superoxide dismutase; TOMM20: translocase of the outer mitochondrial membrane complex subunit 20.

**Table 2:** Mitochondrial dysfunction in genetic models of PAcidemia and MMAcidemia.

Organic Aciduria		Tissue Biochemical and Morphological Mitochondrial Alterations		References
PAcidemia	Mice	Brain	↑ Complex II activity	[54]
			↓ Complex I-III, II-III, III and IV activities	
			↑ MDA, isoprostanes and ROS levels	
		Liver	↑ Complex I, II, II-III, III and IV activities	[54]
			↑ MDA and ROS levels	
			↓ GPx expression	
		Skeletal muscle	↓ Complex III activity	[54]
			↑ MDA levels	
		Heart	↓ Complex I activity	[54]
			↑ MDA and ROS levels	[54]
			Impaired cellular calcium homeostasis	[60]
		Kidney	Alterations in mitochondrial ultrastructure	[57]
			↓ CAC-dependent energy generation	
			↓ Expression of PGC1α, OPA1, Mfn1, Mfn2 and PINK1	
			↑ Expression of Drp1	
	Caenorhabditis elegans		↓ Mitochondrial oxidative phosphorylation	[59]
	HL-1 cells		Oxidative stress induction	[77]
MMAcidemia	Mice	Liver	Abnormally shaped mitochondria with disorganized cristae	[55]
			↓ Complex I-III, II-III and IV activities	[26]
			↓ α-KGDH activity	[56]
			↓ GSH and GSSG levels	[26]
	Zebrafish	Kidney	Altered mitochondrial morphology	[31]
			↓ Mitochondrial bioenergetics	
		Liver	Altered mitochondrial morphology	[31]
			↓ Mitochondrial bioenergetics	

CAC: citric acid cycle; Drp1: dynamin-related protein 1; GSH: reduced glutathione; GSSG: oxidized glutathione; HL-1: cardiac muscle cell line; α-KGDH: α-ketoglutarate dehydrogenase; MDA: malondialdehyde; Mfn1: mitofusina 1; Mfn2: mitofusina 2; MMAcidemia: methylmalonic acidemia; OPA1: optic atrophy 1; PAcidemia: propionic acidemia; PGC1α: peroxisome proliferator-activated receptor gamma coactivator 1-α; PINK-1: PTEN-induced putative kinase 1; ROS: reactive oxygen species.

## 4. Role of the Accumulated Metabolites in the Underlying Mechanisms Associated with Mitochondrial Toxicity and Oxidative Stress in MMAcidemia and PAcidemia

Mounting evidence from human studies and animal models supports the hypothesis that metabolite accumulation in PAcidemia and MMAcidemia disrupts multiple processes of mitochondrial function. The accumulated metabolites, particularly MMA, PA, 3-HPA, 2-MCA and MA, have been shown to impair several essential mitochondrial processes, both in vivo and in vitro in rat tissues and in cultured cells. In the following sections, we summarize and discuss these findings, emphasizing the major mitochondrial systems affected by these compounds, namely bioenergetics (Table 3), calcium homeostasis (Table 4), mitochondrial quality control (Table 5) and redox balance (Table 6).

### 4.1. Bioenergetics Dysfunction

MMAs were shown to inhibit the activities of respiratory chain complexes in rat cerebral cortex [78], striatum [79], and hippocampus [79], and in embryonic rat striatal cell cultures [80], as well as succinate dehydrogenase (SDH) and β-hydroxybutyrate dehydrogenase (HBDH) in the forebrain [81]. MMA also impairs mitochondrial metabolic activity, as observed by decreased MTT reduction in rat brain synaptosomes [82], reduced ATP levels and ΔΨm in PC12 (pheochromocytoma cell line) cells [83], and diminished CoQ_10_ levels, complex II-III activity and MTT reduction in SH-SY5Y (human-derived neuroblastoma cell line) cells [84].

Moreover, in vivo administration of MMA decreases Na^+^,K^+^-ATPase activity in rat striatum [85] and cerebral cortex [86], in addition to reducing ATP synthesis and ΔΨm in the hippocampus [83]. In vitro data demonstrated that MMA reduces ATP-linked mitochondrial respiration supported by NADH [87] and FADH_2_ [88], indicating that MMA acts as a metabolic inhibitor capable of impairing ATP synthesis. The MMA-induced reduction in ATP-linked respiration was presumed to be related to inhibition of GDH, α-KGDH, and aspartate transaminase (AST) activities, and associated with enhanced mitochondrial α-ketoglutarate efflux [87]. It was also described that MMA impairs mitochondrial respiration in SH-SY5Y [89] and C6 glioma [90] cells supported by glucose.

In addition to these harmful effects, MMA inhibits mitochondrial succinate uptake via the dicarboxylate carrier, thereby compromising succinate-supported respiration [88]. Finally, mitochondrial creatine kinase (CK), a key enzyme for intracellular energy transfer, was decreased by MMA in the cerebral cortex [91], a defect that may reduce the cellular ATP/ADP ratio, consistent with previous findings in primary neuronal cultures exposed to MMA [92].

On the other hand, PA has been shown to decrease Na^+^,K^+^-ATPase activity in the cerebral cortex [93], as well as MTT reduction in brain synaptosomes [82], reflecting bioenergetics impairment. Furthermore, MA, and PA to a lesser extent, were demonstrated to impair mitochondrial respiratory activity and ATP generation in rat kidney [94,95] and heart [96] mitochondria. MA-induced alterations were partly due to inhibition of α-KGDH and GDH activities [94], in addition to CoA depletion [96]. MA also decreased MTT reduction and mitochondrial respiration in HEK-293 (Human Embryonic Kidney 293) [95] and H9c2 (H9c2 rat cardiomyoblast) [96] cells.

Finally, 2MCA, which accumulates in both MMAcidemia and PAcidemia, markedly disrupts several mitochondrial functions in the brain. It behaves as a metabolic inhibitor of mitochondrial glutamate oxidation due to GDH inhibition, leading to a decrease in ATP-linked (ADP-stimulated) and uncoupled (CCCP-stimulated) respiration, thereby impairing ATP synthesis [97].

**Table 3:** Metabolite accumulation in PAcidemia and MMAcidemia disrupts mitochondrial bioenergetics in vitro and in vivo in rat tissues and in cultured cells.

Organic Acid		Tissue	Disruption of Mitochondrial Bioenergetics	References
MMA	In vitro	Brain	↓ MTT reduction (mitochondrial function)	[82]
			↓ SDH and HBDH activities	[81]
			↓ ATP-linked mitochondrial respiration	[87,88]
			↓ Mitochondrial succinate uptake	[88]
			↓ GDH, α-KGDH and AST activities	[87]
		Cerebral cortex	↓ Complex II and II-III activities	[78]
			↓ Mi-CK activity	[91]
		Striatum	↓ Complex II and II-III activities	[79,80]
			↓ ATP/ADP ratio	[92]
		Hippocampus	↓ Complex II and II-III activities	[79]
		SH-SY5Y cells	Impaired mitochondrial respiration	[89]
		C6 glioma	Impaired mitochondrial respiration	[90]
		PC12 cells	↓ ATP levels	[83]
			↓ ΔΨm	
		SH-SY5Y cells	↓ CoQ_10_ levels	[84]
			↓ Complex II-III activity	
			↓ MTT reduction (mitochondrial function)	
	Intrastriatal injection	Striatum	↓ Na^+^, K^+^-ATPase activity	[85]
	Subcutaneous injection	Cerebral cortex	↓ Na^+^, K^+^-ATPase activity	[86]
		Hippocampus	↓ ATP levels	[83]
			↓ ΔΨm	
PA	In vitro	Brain	↓ MTT reduction (mitochondrial function)	[82]
		Cerebral cortex	↓ Na^+^, K^+^-ATPase activity	[93]
		Heart	↓ Mitochondrial respiration	[96]
			↓ ATP generation	
		Kidney	↓ Mitochondrial respiration	[94]
			↓ ATP generation	[95]
2MCA	In vitro	Brain	↓ ATP-linked and maximal mitochondrial respiration	[97]
			↓ ATP synthesis	
			↓ GDH activity	
MA	In vitro	Heart	↓ Mitochondrial respiration	[96]
			↑ CoA depletion	
			↓ KGDH activity	
			↓ ATP generation	
		H9c2 cells	↓ Mitochondrial respiration	[96]
		Kidney	↓ Mitochondrial respiration	[94]
			↓ KGDH and GDH activities	[94]
			↓ ATP generation	[95]
		HEK-293 cells	↓ Mitochondrial respiration	[94]
			↓ MTT reduction (mitochondrial function)	[95]

AST: aspartate transaminase; CoQ_10_: coenzyme Q_10_; GDH: glutamate dehydrogenase; H9c2: H9c2 rat cardiomyoblast; HBDH: β-hydroxybutyrate dehydrogenase; HEK-293: Human Embryonic Kidney 293; α-KGDH: α-ketoglutarate dehydrogenase; 2MCA: 2-methylcitric acid; MA: maleic acid; Mi-CK: mitochondrial creatine kinase; MMA: methylmalonic acid; MMAcidemia: methylmalonic acidemia; MTT: 3-(4,5-dimethylthiazol-2-yl)-2,5-diphenyltetrazolium bromide; PA: propionic acid; PAcidemia: propionic acidemia; PC12: pheochromocytoma cell line; SDH: succinate dehydrogenase; SH-SY5Y: human-derived neuroblastoma cell line.

**Table 4:** Metabolite accumulation in PAcidemia and MMAcidemia disrupts mitochondrial calcium homeostasis in vitro in rat tissues.

Organic Acid		Tissue	Disruption of Mitochondrial Calcium Homeostasis	References
MMA	In vitro	Brain	↓ ΔΨm and matrix NAD(P)H content in Ca^2+^-loaded mitochondria ↑ Mitochondrial swelling in Ca^2+^-loaded mitochondria ↑ Ca^2+^-induced mPT pore opening	[98]
PA	In vitro	Heart	↓ ΔΨm in Ca^2+^-loaded mitochondria ↑ Mitochondrial swelling in Ca^2+^-loaded mitochondria ↑ Ca^2+^-induced mPT pore opening	[96]
2MCA	In vitro	Brain	↓ ΔΨm in Ca^2+^-loaded mitochondria ↑ Mitochondrial swelling in Ca^2+^-loaded mitochondria ↑ Ca^2+^-induced mPT pore opening	[97]
MA	In vitro	Heart	↓ ΔΨm and matrix NAD(P)H content in Ca^2+^-loaded mitochondria ↑ Mitochondrial swelling in Ca^2+^-loaded mitochondria ↑ Ca^2+^-induced mPT pore opening ↓ Ca^2+^ retention capacity	[96]
		Kidney	↓ ΔΨm and matrix NAD(P)H content in Ca^2+^-loaded mitochondria ↑ Mitochondrial swelling in Ca^2+^-loaded mitochondria ↑ Ca^2+^-induced mPT pore opening ↓ Ca^2+^ retention capacity	[95]

Ca^2+^: calcium; 2MCA: 2-methylcitric acid; MA: maleic acid; MMA: methylmalonic acid; MMAcidemia: methylmalonic acidemia; mPT: mitochondrial permeability transition; PA: propionic acid; PAcidemia: propionic acidemia.

**Table 5:** Metabolite accumulation in PAcidemia and MMAcidemia disrupts mitochondrial quality control in vitro and in vivo in rat tissues and in cultured cells.

Organic Acid		Tissue	Disruption of Mitochondrial Quality Control	References
MMA	In vitro	PC12 cells	↓ PINK1 and Parkin protein levels	[83]
	Subcutaneous injection	Hippocampus	↓ PINK1 and Parkin protein levels	[83]
PA	In vitro	SH-SY5Y cells	↓ STOML, TFAM, NRF-1 and OPA1 mRNA expression	[99,100]
			↑ PGC1-α and SIRT3 mRNA expression	[100]
			↓ TFAM and SIRT1 mRNA expression	[100]
			↑ Mitophagy	[101]

MMA: methylmalonic acid; MMAcidemia: methylmalonic acidemia; NRF-1: nuclear respiratory factor 1; OPA1: optic atrophy 1; PA: propionic acid; PAcidemia: propionic acidemia; PC12: pheochromocytoma cell line; PGC1-α: peroxisome proliferator-activated receptor gamma coactivator 1-α; PINK-1: PTEN-induced putative kinase 1; SH-SY5Y: human-derived neuroblastoma cell line; SIRT1: sirtuin 1; SIRT3: sirtuin 3; STOML: stomatin-like protein; TFAM: transcription factor A.

### 4.2. Disruption of Calcium Homeostasis

MMA was shown to decrease succinate-supported respiration and induce mPT in Ca^2+^-loaded brain mitochondria, leading to ΔΨm collapse, a decrease in matrix NAD(P)H content and mitochondrial swelling [98]. In addition, MA severely disturbs Ca^2+^ homeostasis in rat heart [96] and kidney [95] mitochondria, leading to a collapse of mitochondrial functions associated with mPT induction, including ΔΨm depolarization, a decrease in NAD(P)H pool, swelling induction and reduction of Ca^2+^ retention capacity. Similar but less intense effects were observed for PA. 2MCA, which accumulates in both MMAcidemia and PAcidemia, was also found to induce mPT pore opening, loss of ΔΨm, and mitochondrial swelling in Ca^2+^-loaded brain mitochondria [97].

### 4.3. Impairment of Mitochondrial Quality Control

Recent data have shown that PA and MMA not only disturb mitochondrial functions but also disrupt mitochondrial quality control processes, including biogenesis, dynamics, and mitophagy.

Thus, PA exposure to SH-SY5Y cells led to smaller, structurally altered mitochondria and changes in fusion/fission-related dynamics proteins. Specifically, PA decreased the mRNA expression of stomatin-like protein (STOML), transcription factor A (TFAM), nuclear respiratory factor 1 (NRF-1), and OPA1 [99]. This organic acid also increased mtDNA copy number and altered the mRNA expression of markers of mitochondrial biogenesis, including an increase in PGC1-α and SIRT3 and a decrease in TFAM and SIRT1 [100]. Together, these changes are consistent with a compensatory response to damage. It should be noted, however, that the protective value of compensatory mechanisms is likely transient. The transition from a compensatory to a maladaptive state is expected to occur when the rate of PA-induced mitochondrial injury exceeds the turnover capacity of the quality control machinery, i.e., when the sustained demand for mitophagy and biogenesis is no longer able to compensate for accumulating organelle damage and to replenish functional mitochondria. This notion is corroborated by a recent study evidencing that PA decreased viability of SH-SY5Y cells despite active mitophagy [101]. Taken together, these results support the notion that PA triggers mitochondrial injury while simultaneously engaging adaptive mitochondrial quality control pathways.

By contrast, MMA was shown to disrupt mitochondrial quality control processes, especially at the level of PINK1/Parkin-dependent mitophagy, instead of promoting adaptative responses. In the hippocampus of rats subjected to subcutaneous injection of MMA and in MMA-treated PC12 cells, this organic acid decreased the expression of the mitophagy-related proteins PINK1 and Parkin, indicating inefficient clearance of damaged mitochondria [83]. Indeed, mitochondrial structural damage, oxidative stress, reduced ATP levels and lower ΔΨm were also found in the hippocampus of these animals [83].

Overall, these findings converge on the view that PA- and MMA-induced mitochondrial dysfunction is closely related to changes in mitochondrial quality control. Thus, PA, provokes stress-induced remodeling and compensatory turnover, which may shift from an adaptive to a maladaptive state when injury outpaces clearance capacity [99,100,101], whereas MMA markedly impairs effective mitochondrial clearance, disrupting the quality control response at its onset rather than overwhelming it [83].

### 4.4. Oxidative Stress

In vitro studies demonstrate that the metabolites accumulating in MMAcidemia and PAcidemia induce cellular and molecular oxidative damage. In this particular, since reduced electron flux through the mitochondrial respiratory chain is associated with increased production of ROS and RNS [64,65], inhibition of respiratory chain complex activities by MMA is expected to contribute to oxidative stress, although other cellular sources of reactive species exist. Multiple studies have shown that MMA disrupts redox balance both in vitro and in vivo in the rat brain. MMA induces lipid peroxidation (increases in chemiluminescence and thiobarbituric acid-reactive substances—TBA-RS—levels) and decreases antioxidant defenses (total radical-trapping antioxidant potential—TRAP) in the brain [102]. Elevated TBA-RS (lipid peroxidation), increased protein carbonyl content (protein oxidation), enhanced DCFH oxidation (ROS generation), and reduced GPx activity by MMA have been demonstrated in brain neuronal terminal synaptosomes [103]. More recently, MMA was shown to increase ROS and MDA levels and decrease SOD activity in PC12 cells [83].

On the other hand, intrastriatal injection of MMA leads to increased protein carbonylation and elevated TBA-RS levels [85,104,105], whereas subcutaneous administration produces lipid (increased TBA-RS), protein (increased carbonyl) and DNA oxidative damage, along with decreased SOD activity in the cerebral cortex and kidney of rats [106,107]. A more recent study also demonstrated increased MDA and ROS levels, associated with a decrease in SOD activity in the hippocampus of rats subjected to subcutaneous administration of MMA [83]. Furthermore, a single neonatal intracerebroventricular injection of MMA was shown to increase DCFH oxidation (ROS production) in the cerebral cortex and striatum, accompanied by elevated inflammatory cytokines and apoptotic markers [108].

In regards to PA, it was demonstrated that this organic acid induced lipoperoxidation (increases in chemiluminescence and TBA-RS levels) while decreasing TRAP in brain tissue in vitro [102]. Decreased TRAP has also been documented in the hippocampus following chronic subcutaneous administration of PA [109], and protein carbonylation increases after intrastriatal PA injection [110]. Another study reported that both MMA and PA induced ROS generation and lipid oxidative damage in brain synaptosomes [82]. Finally, it was reported that MMA, PA, 2MCA, 3HPA and PG caused significant DNA oxidative damage in human peripheral leukocytes [111,112].

**Table 6:** Metabolite accumulation in PAcidemia and MMAcidemia disrupts mitochondrial redox balance in vitro and in vivo in rat tissues and in cultured cells.

Organic Acid		Tissue	Disruption of Mitochondrial Redox Balance	References
MMA	In vitro	Brain	↑ Chemiluminescence and TBA-RS levels	[82,102]
			↑ Carbonyl formation	[103]
			↑ DCFH oxidation	[82,103]
			↓ GPx activity	[103]
			↓ TRAP	[102]
		Leukocytes	↑ DNA oxidative damage	[111,112]
		PC12 cells	↑ MDA levels	[83]
			↑ ROS levels	
			↓ SOD activity	
	Intrastriatal injection	Striatum	↑ TBA-RS levels	[85,105]
			↑ Carbonyl formation	[104,105]
	Intracerebroventricular injection	Cerebral cortex	↑ DCFH oxidation	[108]
		Striatum	↑ DCFH oxidation	[108]
	Subcutaneous injection	Cerebral cortex	↑ TBA-RS levels and carbonyl content	[106]
			↑ DNA oxidative damage	[107]
			↓ SOD activity	[106]
		Hippocampus	↑ MDA levels	[83]
			↑ ROS levels	
			↓ SOD activity	
		Kidney	↑ TBA-RS levels and carbonyl content	[106]
			↑ DNA oxidative damage	[107]
			↓ SOD activity	[106]
PA	In vitro	Brain	↑ Chemiluminescence and TBA-RS levels	[82,102]
			↓ TRAP	[102]
			↑ DCFH oxidation	[82]
		Leukocytes	↑ DNA oxidative damage	[111,112]
	Intrastriatal injection	Striatum	↑ Carbonyl formation	[110]
	Subcutaneous administration	Hippocampus	↓ TRAP	[109]
2MCA	In vitro	Leukocytes	↑ DNA oxidative damage	[112]
3HPA	In vitro	Leukocytes	↑ DNA oxidative damage	[112]
PG	In vitro	Leukocytes	↑ DNA oxidative damage	[112]

DCFH: 2′,7′-dichlorofluorescein; GPx: glutathione peroxidase; 3HPA: 3-hydroxypropionic acid; 2MCA: 2-methylcitric acid; MDA: malondialdehyde; MMA: methylmalonic acid; PA: propionic acid; PC12: pheochromocytoma cell line; PG: propionylglycine; ROS: reactive oxygen species; SOD: superoxide dismutase; TBA-RS: thiobarbituric acid-reactive substances; TRAP: total radical-trapping antioxidant capacity.

## 5. Concluding Remarks

**Figure 2:**
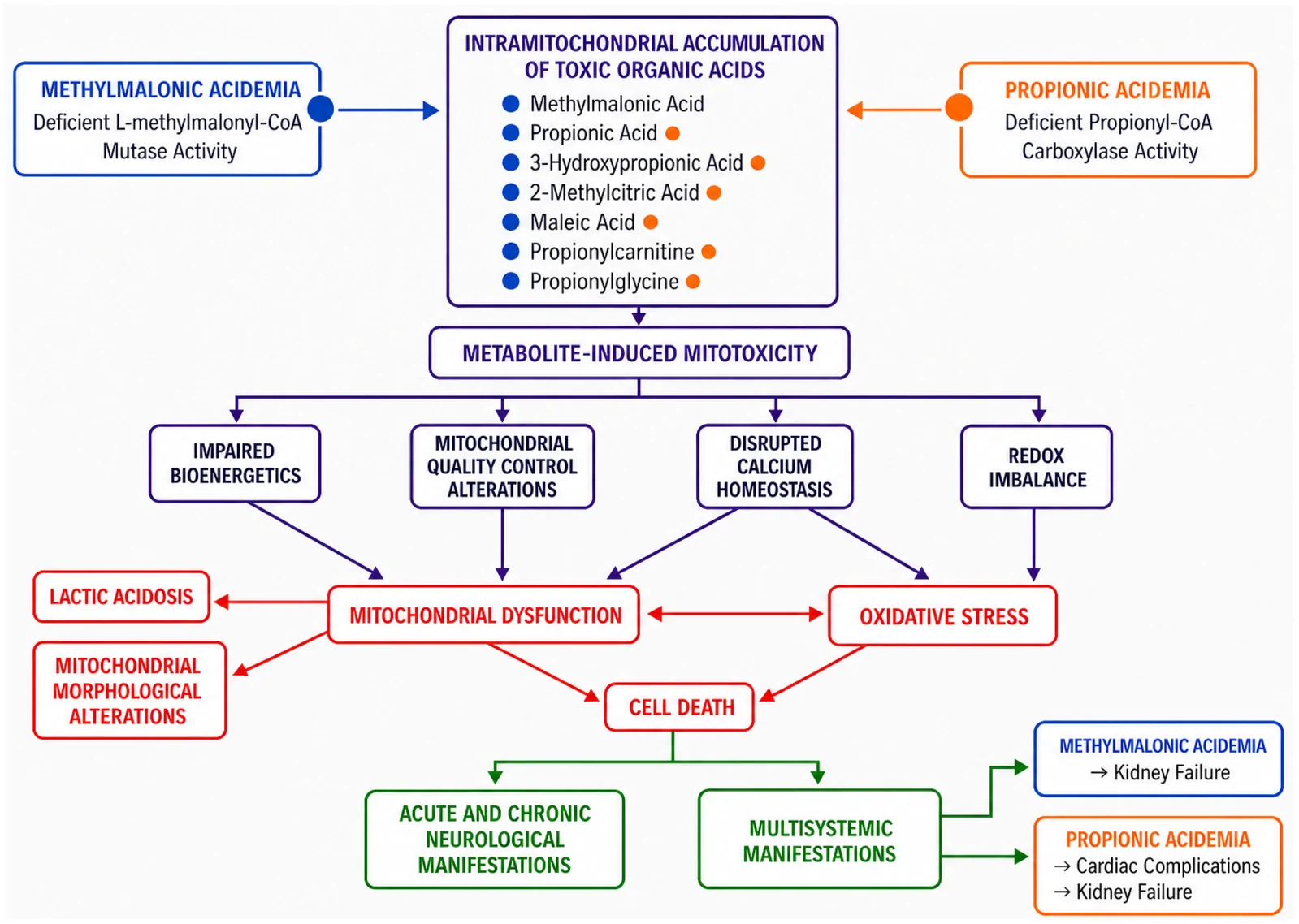
Proposed mechanisms of mitotoxicity induced by metabolites accumulating in methylmalonic acidemia and propionic acidemia. Methylmalonic acid, propionic acid, 3-hydroxypropionic acid, 2-methylcitric acid, maleic acid, propionylcarnitine and/or propionylglycine accumulate in tissues and biological fluids because of impaired propionate catabolism. These metabolites compromise mitochondrial bioenergetics, mitochondrial quality control, calcium homeostasis and redox balance, leading to mitochondrial dysfunction and oxidative stress, and ultimately to cell death. The resulting cellular injury contributes to the multisystemic manifestations of both disorders. Figure created with Canva web application (Canva Pty Ltd., Sydney, Australia).

A great deal of evidence has revealed that the major metabolites accumulating in MMAcidemia and PAcidemia disrupt essential mitochondrial systems, including bioenergetics, calcium homeostasis, and mitochondrial dynamics, in addition to inducing oxidative stress (Figure 2). These findings are consistent with observations obtained in human patients and genetic models of these diseases, strongly indicating that metabolite-induced mitochondrial dysfunction and altered redox homeostasis represent central pathomechanisms contributing to tissue damage in these disorders. Importantly, a key limitation is that most research on metabolite toxicity has not investigated potential synergistic or antagonistic effects, making it difficult to draw conclusions about their interactions in these diseases.

Although experimental evidence indicates that these mitochondrial disturbances are secondary to the mitotoxicity caused by the metabolites that accumulate in these disorders, it does not precisely define the primary insult. Nevertheless, a primary bioenergetics insult induced by these accumulating metabolites, resulting in impaired ATP generation, may be related to the disruption of mitochondrial calcium homeostasis, quality control, and redox balance [113].

Consequently, in addition to current therapeutic approaches, such as dietary protein restriction and L-carnitine supplementation, the development of new strategies aimed at improving mitochondrial functions may offer significant benefits for attenuating disease progression and improving clinical outcomes in MMAcidemia and PAcidemia. However, their clinical translation faces considerable barriers, including the efficient delivery of therapeutic compounds to target tissues, particularly across the blood–brain barrier, and the risk of off-target effects that may limit their safety and efficacy.

In this regard, preclinical studies have demonstrated that supplementing hepatocytes from MMAcidemia and PAcidemia individuals with disodium citrate [114], and treating MMAcidemia patient-derived fibroblasts with dimethyl-oxoglutarate [115], increased CAC intermediates, supporting mitochondrial energy production. Furthermore, bezafibrate, a PPAR agonist that upregulates PGC1-α-dependent mitochondrial biogenesis, was shown to attenuate MMA-induced mitotoxicity in C6 glioma cells [90], whereas intraperitoneal creatine injection, leading to increased ATP levels via the phosphocreatine/CK pathway, protected against MMA-induced seizures and oxidative damage following intrastriatal MMA injection in rats [105]. In addition, oral treatment with the antioxidants MitoQ or resveratrol ameliorated tissue oxidative damage in the mouse genetic model [116] and in fibroblasts from PAcidemia patients [117], reinforcing the role of oxidative stress as a pathomechanism of tissue damage in this disorder. Furthermore, CoQ_10_ supplementation in the MMAcidemia mouse model ameliorated the glomerular filtration rate, reflecting improved renal function [30]. On the other hand, the sesquiterpenoid compound costunolide promoted mitophagy and mitigated oxidative stress via the PINK1/Parkin pathway in the brain of rats subjected to a chemically induced MMAcidemia model achieved by intraperitoneal injection of MMA, in addition to mitigating cognitive impairment in these animals [83]. Finally, a clinical study demonstrated that citrate supplementation in PAcidemia patients increased the urinary excretion of CAC intermediates, although it did not mitigate motor and cognitive developmental delay [118]. It is, however, stressed that these improvements still need to be confirmed in controlled clinical trials before they can be considered as effective alternative therapies for MMAcidemia and PAcidemia patients.

## Data Availability

No new data were created or analyzed in this study. Data sharing is not applicable to this article.

## Abbreviations

The following abbreviations are used in this manuscript:

AST	Aspartate aminotransferase
CAC	Citric acid cycle
CAT	Catalase
CK	Creatine kinase
CoQ_10_	Coenzyme Q_10_
DCFH	2′,7′-dichlorofluorescein
Drp1	Dynamin-related protein 1
ER	Endoplasmic reticulum
GDH	Glutamate dehydrogenase
GPDH	Glycerol-3-phosphate dehydrogenase
GPx	Glutathione peroxidase
GR	Glutathione reductase
GSH	Reduced glutathione
GSSG	Oxidized glutathione
GST	Glutathione S-transferase
H9c2	H9c2 rat cardiomyoblast
HBDH	β-Hydroxybutyrate dehydrogenase
HEK-293	Human Embryonic Kidney 293
HL-1	Cardiac muscle cell line
IDH	Isocitrate dehydrogenase
iPSC	Induced pluripotent stem cells
α-KGDH	α-Ketoglutarate dehydrogenase
MA	Maleic acid
2MCA	2-Methylcitric acid
MDA	Malondialdehyde
Mfn1	Mitofusin 1
Mfn2	Mitofusin 2
MMA	Methylmalonic acid
MMAcidemia	Methylmalonic acidemia
ΔΨm	Mitochondrial membrane potential
mPT	Mitochondrial permeability transition
NRF-1	Nuclear respiratory factor 1
OPA1	Optic atrophy 1
PA	Propionic acid
PAcidemia	Propionic acidemia
PC12	Pheochromocytoma cell line
PDH	Pyruvate dehydrogenase
PGC1-α	Peroxisome proliferator-activated receptor gamma coactivator 1-alpha
Pi	Inorganic phosphate
PINK1	PTEN-induced putative kinase 1
PG	Propionylglycine
RNS	Reactive nitrogen species
ROS	Reactive oxygen species
SR	Sarcoplasmic reticulum
SDH	Succinate dehydrogenase
SH-SY5Y	Human-derived neuroblastoma cell line
SIRT1	Sirtuin 1
SIRT3	Sirtuin 3
SOD	Superoxide dismutase
STOML	Stomatin-like protein
TBA-RS	Thiobarbituric acid-reactive substances
TFAM	Transcription factor
TOMM20	Translocase of the outer mitochondrial membrane complex subunit 20
TRAP	Total radical-trapping antioxidant capacity
